# The muscarinic agonist arecoline suppresses motor circuit hyperactivity in *C. elegans*

**DOI:** 10.17912/micropub.biology.000272

**Published:** 2020-06-26

**Authors:** Katherine A McCulloch, Yishi Jin

**Affiliations:** 1 Neurobiology Section, Division of Biological Sciences, University of California San Diego, La Jolla, CA 92093

**Figure 1:**
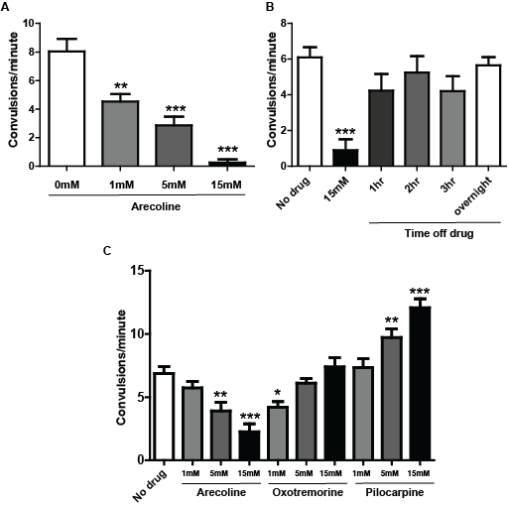
(A) Shown is the convulsion rate for *acr-2(gf)* animals cultured on nematode growth media (NGM) supplemented with 1mM, 5mM, or 15mM arecoline, compared to no drug control. (B) Shown is the convulsion rate of *acr-2(gf)* animals treated with 15mM arecoline on NGM plates for 3hrs, then transferred to drug-free plates, and scored after the indicated time post-arecoline treatment. (C) Varied effects of cholinergic agonists on convulsions of *acr-2(gf)* animals. Convulsions were scored after 3hr of indicated drug treatment. N≥18 * P≤0.05, **P≤0.01, **P≤0.001 Two-way ANOVA followed by Dunnett’s Test. All treatments are compared to those on drug-free NGM plates.

## Description

Balanced excitation and inhibition activities are critical for proper neural circuit function. The *C. elegans* locomotor circuit is regulated via the coordinated activities of cholinergic excitation and GABAergic inhibition that promote muscle contraction and relaxation, respectively. A gain-of-function mutation in a neuronal acetylcholine receptor subunit gene, *acr-2* [*acr-2(gf)*], causes both increased cholinergic excitation and decreased GABAergic inhibition to body muscles, resulting in overall hyperactivity of the locomotor circuit. *acr-2(gf)* mutant animals show Uncoordinated(Unc) movement and spontaneous shrinking, or, convulsion (Jospin **et al.** 2009).

Acetylcholine activates nicotinic receptors, which are ion channels that mediate fast synaptic transmission, and muscarinic receptors, which are G-protein coupled receptors that modulate neuronal activity via intracellular signaling (Albuquerque **et al.** 2009; Jones **et al.** 2012). As implied by their names, these receptors have different pharmacology that distinguish their activities. Similar to nicotine, the muscarinic agonist arecoline is used as a recreational drug, particularly in Asia, where it is often chewed in a preparation from the betel quid plant known as paan (WHO 2004). In *C. elegans*, arecoline has been used to study muscarinic functions in pharyngeal pumping and the motor circuit, where muscarinic signaling serves a modulatory role (Lackner **et al.** 1999; Robatzek **et al.** 2001; Steger and Avery 2004). Wild-type animals exposed to arecoline have slightly elevated cholinergic release, exhibiting, for example, faster locomotion and increased abundance and activity of synaptic proteins in cholinergic neurons (Chan **et al.** 2012; Chan and Sieburth 2012; Lackner **et al.** 1999).

Previous studies from our lab found that loss of function mutations in *sphk-1,* a lipid kinase, can suppress *acr-2(gf)* phenotypes (McCulloch **et al.** 2017). *sphk-1* is an effector for muscarinic signaling (Chan **et al.** 2012; Chan and Sieburth 2012). Based on the interaction between *acr-2(gf)* and *sphk-1*, we tested the effect of arecoline on *acr-2(gf)* mutant animals. As arecoline is a cholinergic agonist and may likely induce parallel elevation of cholinergic signaling, we expected to observe an enhancement of *acr-2(gf)* phenotypes. However, to our surprise, arecoline treatment strongly suppressed the convulsion rate of *acr-2(gf)* animals*.* We observed a dose-dependent suppression of convulsion after 3hrs of arecoline treatment (Figure 1A). The Unc phenotype of these animals was also strongly suppressed, with animals moving rapidly across the plate following drug treatment. This effect of arecoline is reversible, and the convulsion phenotype almost fully recovered after 1hr off drug (Figure 1B).

Arecoline is one of many muscarinic agonists that have been identified and used in pharmacological studies of cholinergic signaling, and different muscarinic agonists can have preferential effects on different receptor subtypes. Arecoline is a relatively non-specific muscarinic agonist, able to act through all of the vertebrate subtypes (Rang *et al*. 2012). Additionally, arecoline has been shown to activate nicotinic receptors, although with much less potency (Papke **et al.** 2015). We next wanted to determine if the observed arecoline effect was a general property of muscarinic agonists. We tested two other drugs, pilocarpine and oxotremorine, which have specificity for the excitatory M1 muscarinic receptor subtype. For example, pilocarpine has been used for decades to induce frontal temporal lobe seizure in murine models, and this action is via the M1 receptor in the brain (Hamilton **et al.** 1997). Oxotremorine is also a highly specific muscarinic agonist (Rang *et al*. 2012). We observed that these muscarinic agonists showed varied effects on *acr-2(gf)* behaviors. Pilocarpine treatment enhanced *acr-2(gf)* in a dose-dependent manner, which would be consistent with this drug stimulating cholinergic activity. In contrast, Oxotremorine had an effect more similar to arecoline, although suppression was only observed at the lowest concentration tested, 1mM (Figure 1C).

Together, these data show that, in the context of *acr-2(gf)* induced circuit hyperactivity, cholinergic agonists can have varied effects, and imply additional targets of these drugs in *C. elegans*. Future studies will involve identifying the pathways and receptors that mediate these different functions.

## Methods

Drug plates were prepared by supplementing standard NGM plates with drugs essentially as described for other *C. elegans* pharmacology assays (Mahoney **et al.** 2006). Drugs were dissolved in NGM at indicated concentrations prior to pouring. NGM-only plates were used as no-drug controls. All plates were seeded with a thin lawn of OP50 bacterial food. Two trials were performed for each experiment, with typically 10 animals in each trial. Muscarinic agonists often drive animals to crawl off the plates, especially for those that suppress *acr-2(gf)*, so some trials had <10 animals when scored. Animals were transferred to drug plates and then scored after 3 hours. Convulsions were counted over 90s, and then normalized to 60s as convulsions per minute.

## Reagents

STRAINS

**MT6241** *acr-2(n2420gf)* X

Drugs used in this study:

Arecoline hydrobromide, Acros Organics Cat#AC250130050

Oxotremorine M, Sigma Aldrich Cat#0100-500MG

Pilocarpine hydrochloride, Fisher Scientific Cat#ICN15189210
